# Vitiligo responds to topical aryl hydrocarbon receptor agonist tapinarof (WBI-1001)

**DOI:** 10.1016/j.jdcr.2023.06.051

**Published:** 2023-07-26

**Authors:** Lisa Liu, Qianli Yang, Ming-wan Su, Youwen Zhou

**Affiliations:** a Department of Dermatology and Skin Science, University of British Columbia, Vancouver, BC, Canada; b Department of Dermatology, Huashan Hospital, Fudan University, Shanghai, China

**Keywords:** autoimmune disorder, pigmentation, vitiligo

## Introduction

Vitiligo is an autoimmune disease in which the melanocytes die because of immune cytotoxicity or oxidative stress-induced cell death.^1^ Repigmentation can be achieved with immunosuppressive therapies, such as topical tacrolimus, topical ruxolitinib, and narrow-band UV therapies. However, the repigmentation often takes a long time, and only a minority of patients achieve satisfactory repigmentation. Therefore, there is a need to expand therapeutic options for this disease.

Tapinarof (WBI-1001) is a topical nonsteroidal immune modulating agent, first developed by Welichem (Welichem Biotech Inc.).^2^ It has been demonstrated to be effective in humans for treatment of atopic dermatitis^2^^,^^3^ and psoriasis.^3^, ^4^, ^5^, ^6^ It has recently received regulatory approval as a topical psoriasis therapy in China (2019) and the United States (2022). It is an agonist of aryl hydrocarbon receptor,^7^ which is involved in suppressing immune response and inhibiting oxidative stress. Therefore, in theory, tapinarof may have beneficial effects on vitiligo as it can block the 2 mechanisms for melanocyte death in vitiligo.^1^ Here we report the case of a patient with facial vitiligo who achieved significant repigmentation after using topical tapinarof 1% cream.

## Case description

**Fig 1:**
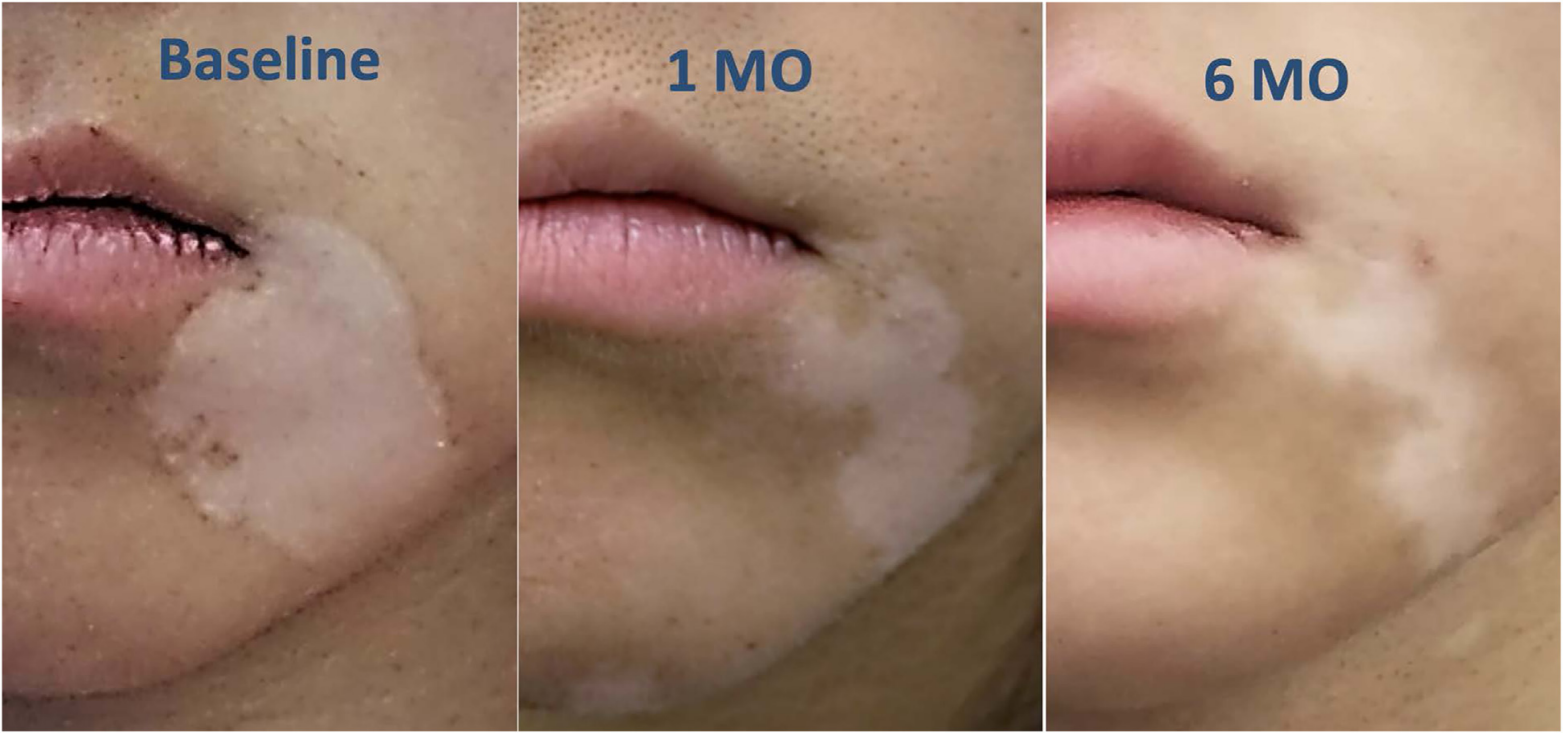
A 19-year-old female patient with vitiligo on the left cheek before receiving the treatment, 1 month after using tapinarof 1% cream, and 6 months after using tapinarof 1% cream.

A 19-year-old female student presented with a white patch on the left cheek for 3 years. She was initially prescribed topical tacrolimus 0.1% ointment twice a day. However, due to strong burning sensation, she could not tolerate this therapy; thus, she was only managing to use it sporadically. The patch continued to expand. Tapinarof 1% cream was then tried off-label for the vitiligo patches. She applied tapinarof 1% cream twice a day without experiencing adverse reactions. At 1 month, she noted signs of repigmentation from the edge, and achieved more than 50% repigmentation by 6 months of treatment (Fig 1).

## Discussion

Tapinarof, also known as benvitimod and WBI1001, is a new topical immune modulating drug approved for the treatment of psoriasis by State Food and Drug Administration (China, 2019) and Food and Drug Administration (the United States, 2022). Moreover, in Phase I/II clinical trials, it has demonstrated efficacy for the treatment of atopic dermatitis.^2^^,^^3^ Our case report demonstrates that tapinarof may be useful for the treatment of vitiligo as well.

**Fig 2:**
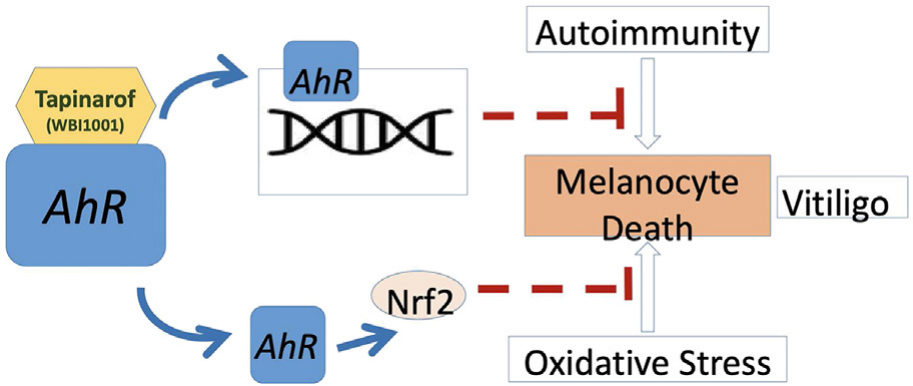
Possible mechanism of action of tapinarof for vitiligo repigmentation. Tapinarof binds to aryl hydrocarbon receptor, resulting in its activation and downstream suppression of immune cytokines and Nrf2-mediated suppression of oxidative stress responses, with the net effect of reduction of melanocyte death in cases of vitiligo.

Tapinarof has been demonstrated to suppress immune active cytokines as well as oxidative stress by inducing expression of Nrf2.^7^ Since these 2 pathways have been proposed to be involved in death of melanocytes in vitiligo,^1^ it was not unexpected that tapinarof may be of benefit for patients with vitiligo (Fig 2). Since topical tapinarof can induce long term remission of psoriasis^8^ and has the ability to reduce formation of memory T cells,^9^ which are involved in vitiligo pathogenesis,^10^ it may also be able to induce long term remission in vitiligo. A randomized double-blind clinical trial is needed to fully evaluate its clinical usefulness for vitiligo.

## Conflicts of interest

None disclosed.
